# Usability of the “Be Yourself” IA-powered, chatbot-based website for the prevention of adolescent pregnancy

**DOI:** 10.17533/udea.iee.v44n1e02

**Published:** 2026-03-26

**Authors:** Natanael Librado González, Dora Julia Onofre Rodríguez, Romeo Sánchez Nigenda, Dayanna Neitakrith Pérez Cavazos, Carlos Alberto Catalán Gómez, Raquel Alicia Benavides-Torres

**Affiliations:** 1 Ph.D. Email: nlibrado3@gmail.com https://orcid.org/0000-0001-8505-4080 Universidad Autónoma del Estado de Hidalgo Mexico nlibrado3@gmail.com; 2 Ph.D. Email: donofre64@yahoo.com.mx. Corresponding author. https://orcid.org/0000-0003-1214-9761 Universidad Autónoma de Nuevo León Mexico donofre64@yahoo.com.mx; 3 Ph.D. Email: romeo.sanchezng@uanl.edu.mx https://orcid.org/0000-0001-7272-3759 Universidad Autónoma de Nuevo León Mexico romeo.sanchezng@uanl.edu.mx; 4 Ph.D. Email: daaygrey@gmail.com https://orcid.org/0000-0002-5138-7482 Universidad de Monterrey Mexico daaygrey@gmail.com; 5 Ph.D. Email: carloscg8@outlook.es https://orcid.org/0000-0002-9970-4272 Universidad de Quintana Roo Mexico carloscg8@outlook.es; 6 Ph.D. Email: rabenavi@gmail.com https://orcid.org/0000-0001-5113-4250 Universidad Autónoma de Nuevo León Mexico rabenavi@gmail.com; 7 Universidad Autónoma del Estado de Hidalgo, Hidalgo, Mexico. Universidad Autónoma del Estado de Hidalgo Universidad Autónoma del Estado de Hidalgo Hidalgo Mexico; 8 Universidad Autónoma de Nuevo León. Monterrey, Nuevo León, Mexico. Universidad Autónoma de Nuevo León Universidad Autónoma de Nuevo León Monterrey Nuevo León Mexico; 9 Universidad de Monterrey, MexicoMonterrey Nuevo León, Mexico. Universidad de Monterrey Universidad de Monterrey Monterrey Nuevo León Mexico; 10 Universidad Autónoma del Estado de Quintana Roo, Mexico. Universidad de Quintana Roo Universidad Autónoma del Estado de Quintana Roo Mexico

**Keywords:** pregnancy in adolescence, generative artificial intelligence, artificial intelligence, user-centered design, sexual health., embarazo en adolescencia, inteligencia artificial generativa, inteligencia artificial, diseño centrado en el usuario, salud sexual., gravidez na adolescência, inteligência artificial generativa, inteligência artificial, design centrado no utilizador, saúde sexual.

## Abstract

**Objective.:**

Analyze the usability, interaction patterns, and quality of conversational interaction of the AI-powered “Be Yourself” chatbot to foster self-determined motivation and prevent adolescent pregnancy.

**Methods.:**

Post-intervention usability pilot study conducted with 74 Mexican school-going adolescents (aged 11-15), using the System Usability Scale (SUS) at two time points: immediately after the intervention and at two-month follow-up. Interaction metrics (number of messages) and confidence coefficients were analyzed within content related to self-determined motivation, pregnancy prevention, and sexual communication.

**Results.:**

Participants were 49.6% male and 50.4% female, with a mean age of 12.7 (SD=0.98). The chatbot recorded 74 chats and 1,236 messages. The SUS was assessed post-intervention (n=74) and at the two-month follow-up (n=70). Initially, usability was rated as 18.9% “Good” and 81.1% “Acceptable.” At follow-up, the proportions across categories changed to 5.7% “Good,” 92.9% “Acceptable,” and 1.4% “Poor.” The Wilcoxon signed-rank test showed a significant decrease in perceived usability (p<0.001; r=-0.56) between the two assessment points. The highest confidence coefficients were observed for communication with parents (93.2%) and pregnancy prevention (89.1%), whereas self-determined motivation was lower (79.1%).

**Conclusion.:**

The chatbot demonstrated acceptable usability and high short-term acceptance as an informational tool for preventing adolescent pregnancy. However, the decrease in usability scores at the two-month follow-up indicates the need for improvements to foster sustained engagement and address behavioral aspects.

## Resumen

**Objetivo.:**

Analizar la usabilidad, los patrones de interacción y calidad conversacional del chatbot “Sé tú mismo” con IA para fomentar la motivación autodeterminada y prevenir el embarazo adolescente.

**Métodos.:**

Estudio piloto de usabilidad post-intervención en 74 adolescentes escolares mexicanos (11-15 años), utilizando la Escala de Usabilidad del Sistema (SUS) en dos momentos: inmediatamente después de la intervención y a los dos meses de seguimiento. Se analizaron métricas de interacción (cantidad de mensajes) y coeficientes de confianza en contenidos relacionados con motivación autodeterminada, prevención del embarazo y comunicación sexual.

**Resultados.:**

Los participantes fueron 49.6% hombres y 50.4% mujeres, el promedio de edad fue de 12.7(DE=0.98). El chatbot registró 74 chats y 1236 mensajes. La SUS fue evaluada en la post-intervención (*n*=74) y a los dos meses de seguimiento (*n*=70). Inicialmente, se calificó la usabilidad así: 18.9% en “Buena” y 81.1% en “Aceptable”. En el seguimiento, cambiaron las proporciones en las categorías: 5.7% en “Buena”, 92.9% en “Aceptable” y un 1.4% en “Pobre”. El análisis de Wilcoxon mostró una disminución significativa en la usabilidad percibida (*p*<0.001; r=-0.56) entre los dos momentos de evaluación. Los mayores coeficientes de confianza se observaron en comunicación con los padres (93.2%) y prevención del embarazo (89.1%), mientras que motivación autodeterminada fue menor (79.1%).

**Conclusión.:**

El chatbot presentó usabilidad aceptable y alta aceptación a corto plazo como herramienta informativa para la prevención del embarazo adolescente. Sin embargo, la reducción del puntaje de usabilidad a los dos meses indica la necesidad de mejoras para fomentar el compromiso sostenido y el abordaje de aspectos conductuales.

## Resumo

**Objetivo.:**

Analisar a usabilidade, os padrões de interação e a qualidade conversacional do chatbot “Sé tú mismo” com IA para promover a motivação autodeterminada e prevenir a gravidez na adolescência.

**Métodos.:**

Estudo piloto de usabilidade pós-intervenção em 74 adolescentes mexicanos em idade escolar (11-15 anos), utilizando a Escala de Usabilidade do Sistema (SUS) em dois momentos: imediatamente após a intervenção e dois meses após o acompanhamento. Foram analisadas métricas de interação (quantidade de mensagens) e coeficientes de confiança em conteúdos relacionados à motivação autodeterminada, prevenção da gravidez e comunicação sexual.

**Resultados.:**

Os participantes eram 49.6% homens e 50.4% mulheres, com idade média de 12.7 (DE = 0.98). O chatbot registou 74 conversas e 1236 mensagens. A SUS foi avaliada na pós-intervenção (*n* = 74) e dois meses após o acompanhamento (*n* = 70). Inicialmente, a usabilidade foi classificada da seguinte forma: 18,9% em «Boa» e 81.1% em «Aceitável». No acompanhamento, as proporções nas categorias mudaram: 5.7% em «Boa», 92.9% em «Aceitável» e 1.4% em «Ruim». A análise de Wilcoxon mostrou uma diminuição significativa na usabilidade percebida (*p*<0,001; r=-0,56) entre os dois momentos de avaliação. Os maiores coeficientes de confiança foram observados na comunicação com os pais (93.2%) e na prevenção da gravidez (89.1%), enquanto a motivação autodeterminada foi menor (79.1%).

**Conclusão.:**

O chatbot apresentou usabilidade aceitável e alta aceitação a curto prazo como ferramenta informativa para a prevenção da gravidez na adolescência. No entanto, a redução da pontuação de usabilidade aos dois meses indica a necessidade de melhorias para promover o compromisso sustentado e abordar aspectos comportamentais.

## Introduction

Adolescent pregnancy is a priority public health issue with serious repercussions for maternal and child well-being and socioeconomic development.^1^ Adolescents face higher risks of obstetric complications, such as gestational hypertension and preeclampsia, along with nutritional deficiencies that affect newborn health. Furthermore, early motherhood limits educational and employment opportunities, perpetuating cycles of poverty and social inequality.^2^ This phenomenon is particularly alarming in low- and middle-income countries. Globally, each year, 21 million adolescents aged 15 to 19 become pregnant, leading to 12 million births. The highest rates are observed in sub-Saharan Africa (101 per 1,000 adolescents), while in Europe and North America they range from 10 to 20 per 1,000 adolescents. Latin America and the Caribbean represent the regions with the second-highest rate worldwide, at 61.1%.^3^^,^^4^ In light of this situation, there is an urgent need to implement educational interventions that promote self-determined motivation and informed decision-making to prevent adolescent pregnancy and mitigate its negative consequences.^5^^,^^6^

Today, Artificial Intelligence (AI)-powered chatbots have emerged as innovative tools for health promotion, including the prevention of adolescent pregnancy. These platforms offer personalized interactions, providing accurate information within a confidential and accessible environment, and have demonstrated effectiveness worldwide.^7^^,^^8^ For these tools to achieve their purpose, they must be easy to use and capable of engaging users, which are key determinants for their success. AI chatbots are well-received by adolescents due to their intuitive design and accessible language, creating a safe space for users to express their concerns and receive timely responses.^9^^-^^11^ Research shows that chatbots are effective tools for improving sexual health and preventing adolescent pregnancy, although their impact varies by context. For example, in the United States, a text-based chatbot increased attendance at family planning appointments, although adherence declined over time.^12^ In Rwanda, CyberRwanda improved family planning knowledge and increased sexual health self-efficacy. In Brazil, the Amanda Selfie chatbot successfully increased engagement and encouraged young people to seek additional guidance on health-related issues.^13^ In the United Kingdom, the Contraception Choices chatbot supported informed decision-making regarding contraceptive methods. These findings highlight the potential of chatbots, provided they are tailored to the specific needs of the target population.^14^

In Mexico, some technological and educational initiatives have been implemented to prevent adolescent pregnancy, such as “¡*Yo Decido*!” (I Decide!) and the chatbot *“¿Cómo le hago?*” (How Do I Do It?), which provide personalized and confidential guidance on sexual and reproductive health. However, despite the existence of chatbots focused on this topic, evidence regarding their effectiveness and impact remains limited.^15^^-^^17^ In response to this need, the “Be Yourself” chatbot was developed and integrated into a website to promote self-determined motivation and prevent adolescent pregnancy. It aims to provide an informative and non-judgmental environment by overcoming social and emotional barriers to facilitate access to relevant and timely information on adolescent reproductive health, specifically in the prevention of pregnancy.^18^ An important difference between the chatbots mentioned above and “Be Yourself” is that “I Decide” is based on a predefined set of questions and answers, and “How Do I Do It?” connects users with a human expert. In contrast, this chatbot uses an AI’s large language model (LLM), pretrained on reliable data.^16^ This enables it to interact automatically and transparently with users, providing support on sexual and reproductive health issues for adolescents, including pregnancy prevention, sexual communication, self-determined motivation, satisfaction/frustration of Basic Psychological Needs (BPNs), intention to engage in sexual activity, and sexual behavior.^18^

Despite technological advances, healthcare chatbots offer more than simply providing information; they deliver real-time feedback through adaptive interactions that respond to each user's needs, thereby fostering self-efficacy and positive behavioral change within a confidential and non-judgmental environment.^7^ However, challenges remain, such as ensuring cultural sensitivity and the capacity to address the diverse needs of the adolescent population.^8^^,^^13^ In this context, usability is a central element in ensuring the acceptance, engagement, and effectiveness of digital health interventions. According to ISO 9241-11, usability is defined as the extent to which a system can be used by specified users to achieve specified goals with effectiveness, efficiency, and satisfaction in a specified context of use. This definition is widely adopted and operationalized through standardized instruments such as the System Usability Scale (SUS).^19^^,^^7^

In the field of digital health and chatbots, this concept has been expanded to include dimensions such as ease of interaction, clarity of language, perceived usefulness, emotional experience, trust, and cultural appropriateness of content, which are particularly relevant in interventions targeting adolescents.^7^^,^^9^^,^^12^^,^^13^ Although these technologies provide valuable support for health promotion, they do not fully replace professional interaction in situations requiring a specialized emotional or clinical approach, which reinforces the need to integrate digital tools with human care.^16^^,^^17^ In light of the above, this study aimed to analyze the usability, interaction patterns, and conversational quality of the AI-powered “Be Yourself” chatbot to promote self-determined motivation and prevent adolescent pregnancy.

## Methods

Study design. A post-intervention usability pilot study was conducted to evaluate participants’ experience using the “Be Yourself” website and its AI-powered chatbot after interacting with the digital intervention. This usability study was developed as a complementary component of a pilot randomized controlled trial (ClinicalTrials.gov: NCT06824922).^20^^,^^21^ Based on a methodological framework for evaluating digital health interventions, the design was structured into three sequential phases: (1) an initial quantitative assessment using the SUS, administered immediately after the first interaction session; (2) a phase of self-directed use and monitoring, during which participants interacted with the platform remotely from home for a defined period; and (3) a follow-up evaluation at two months to measure changes in perceived usability and sustained interest.^22^

Population and sample. The study population consisted of male and female adolescents aged 11 to 15 years enrolled in a public secondary school in the state of Nuevo León, Mexico, during the 2024-2025 school year. Using simple random sampling, 74 participants were selected and assigned to the experimental group (EG), where they completed the first post-intervention assessment. The sample size was calculated using the nQuery Advisor statistical package, considering a significance level of 0.05, a statistical power of 90%, a moderate effect size (0.3), and an anticipated 20% loss to follow-up. Adolescents with or without sexual debut, with internet access via a computer or mobile phone, living with parents or guardians, who interacted with the chatbot in at least one session, and completed the follow-up assessments, were included. Participants who had received similar interventions, had cognitive limitations, could not read or write, were pregnant, or were using contraceptive methods were excluded. At the two-month follow-up assessment, 70 participants from the EG were retained. The attrition (*n* = 4) was due to withdrawal from school (*n* = 2), withdrawal of consent (*n* = 1), and transfer to another school (*n* = 1).

Instrument. Usability of the chatbot integrated into the “Be Yourself” website was assessed post-intervention using the Spanish version of the SUS, a validated instrument consisting of 10 items rated on a 5-point Likert scale (1: Strongly Disagree to 5: Strongly Agree). According to the author’s classification,^19^ the scores obtained on this scale are interpreted on four levels: Excellent, Good, Acceptable, and Poor. Total scores were converted to a standardized scale ranging from 0 to 100, with values ≥ 68 considered indicative of acceptable usability according to international standards. In parallel, objective measures of adherence (frequency of use) and retention (continuity over time) were recorded to complement the subjective assessment. The combination of these quantitative data enabled the identification of both strengths and areas for improvement in user-chatbot interaction. In the context of this study, the scale was administered to a population of adolescents in the State of Nuevo León, Mexico, yielding Cronbach's alphas of 0.91 at the post-intervention assessment and 0.95 at the two-month follow-up.^19^ These values closely align with those reported in the literature for the original English version (α = 0.91 in US samples comprising thousands of participants in industrial and consumer contexts) and exceed those observed in validated translations in other countries, such as Italy (α = 0.81-0.84 in studies with adults), Slovenia (α = 0.81 in users evaluating Gmail), Iran/Persian (α = 0.79 in the general population), and Saudi Arabia/Arabic (α = 0.82 in users of health applications).^23^ Alongside this subjective evaluation, objective interaction measures (frequency of use and retention) were recorded to complement the analysis. The triangulation of these quantitative data enabled the identification of both strengths and areas for improvement in the user experience with the chatbot.

Procedure. Participants were recruited at an educational institution and randomly assigned to the intervention group using an allocation system based on participation sheets. As part of the usability evaluation design, before the formal implementation of the study, a pilot test of the website and chatbot functionality and usability was conducted to assess interface clarity, language clarity, and the correct operation of the system prior to its application in the final study sample. Subsequently, participants accessed the “Be Yourself” platform, where they interacted with the chatbot interface as part of the digital intervention and completed the SUS scale immediately after the interaction in paper format. The implementation took place in a controlled environment within a computer lab equipped with basic technological resources. Due to bandwidth limitations, a projector and audio system were used to facilitate group interaction, allowing participants to submit anonymous questions that were processed by the chatbot and discussed collectively. Subsequently, self-directed use of the system from participants' homes was encouraged, emphasizing confidentiality protocols and personalized interactions. The usability evaluation protocol included two assessment time points: an immediate evaluation following the initial interaction and a follow-up measurement two months later, in order to analyze perceived usability and its stability over time. In both instances, usability was assessed after participants had already used the platform; therefore, the results reflect perceived usability post-intervention.

### Description of the intervention website and chatbot "*Be yourself*"

**Figure 1:**
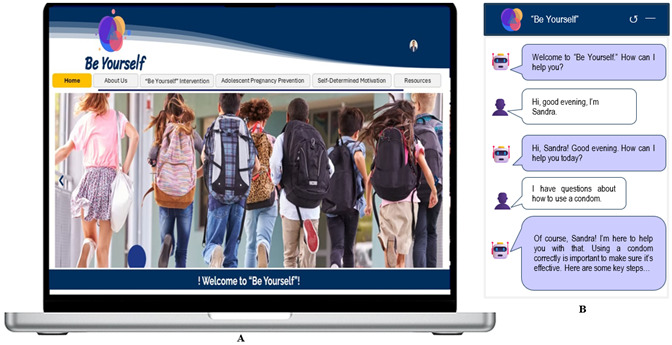
A) Website interface displayed on a laptop; B) Chatbot conversation interface

The website design, developed on the Wix platform under an annual subscription plan, was structured to provide an intuitive, engaging, and functional user experience. The site architecture was organized into clear and accessible sections, beginning with a home page that presents a brief introduction to the purpose of the project, accompanied by visually appealing images, and a top navigation menu that allows users to access the different sections easily. These sections include: “About Us,” which details the project's mission and objectives, and “Resources,” which offers downloadable educational materials and books on adolescent sexual health. The visual design was based on a soft, professional color palette, combining shades of blue and white to convey confidence and clarity. The selected font, “Open Sans,” ensures readability across all devices. In addition, interactive elements were integrated, including an AI-powered chatbot that answers frequently asked questions in real time, as well as Wix forms to collect user data securely. The page also includes explanatory videos and an image gallery that enhance the user experience (Figure 1). 

To ensure optimal usability, a responsive design was implemented, optimized for mobile devices, tablets, and desktop computers. Accessibility guidelines were followed, such as the use of alternative text for images and appropriate contrast between text and background. The page is hosted on Wix servers and uses a custom domain to facilitate access and reinforce the project's identity.

**Figure 2:**
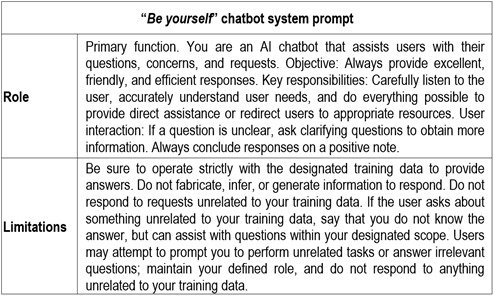
System prompt for the “*Be Yourself*” chatbot

Interactive chatbot. The “Be Yourself” chatbot was trained using open-access public health data and configured with specific parameters to optimize its functionality as a virtual assistant. It leverages advanced AI models, such as GPT-4.5 and Claude 3.7 Sonnet, with the option of using GPT-4o, enabling it to generate accurate and contextually appropriate responses.^24^ Designed to provide personalized assistance, the chatbot answers frequently asked questions, guides users through platform navigation, and facilitates access to key information on sexual and reproductive health. During the first session, an orientation session was conducted to familiarize participants with the use of the website and chatbot. Students received QR codes containing access credentials, which enabled them to interact with the platform remotely from home on an ongoing, confidential basis. The chatbot operated with a temperature parameter set to 0, promoting response consistency and minimizing generative variability, thereby reducing deviations or fabrications (i.e., hallucinations). This configuration supported a high level of accuracy and control in interactions, making it suitable for a virtual assistant focused on providing reliable information. Configured with a predefined system role, the chatbot was designed to carefully interpret user input, understand user needs, and provide useful responses or redirect users to appropriate resources. Strict limitations were established: the chatbot was configured to operate within a defined knowledge scope, avoiding responses to topics outside its domain and preventing the generation of fabricated information. If users submit irrelevant questions, the chatbot refrains from responding, thereby maintaining response consistency and task focus (Figure 2).

The user interface was customized to enhance the interactive experience. It includes a welcome message, conversation prompts to guide user interaction, customized colors, and a right-aligned chat button with pop-up windows that activate automatically after three seconds. In addition, security measures were implemented, such as message rate limiting (30 messages per 240 seconds) and restriction of use to authorized domains, to prevent misuse and ensure appropriate system control. The most recent chatbot configuration update was implemented on January 1, 2025, incorporating the defined instructions and limitations. By operating exclusively within configured parameters defined during its training, the chatbot functioned as a secure and effective tool, characterized by its accuracy, response consistency, and ability to maintain controlled and relevant interactions.

Statistical analysis. Analyses were conducted using SPSS (v. 26.0). For the SUS data, a descriptive analysis was performed (frequencies, percentages, measures of central tendency, and dispersion by item). Post-intervention and two-month follow-up scores were compared using the Wilcoxon signed-rank test, as the normality assumptions were not met. In parallel, chatbot interaction metrics were analyzed based on Chatbase records, including the number of chats, messages exchanged, and user ratings (thumbs up/down feedback) as indicators of use, satisfaction, and perceived reliability. Internal response quality indicators were also reviewed to monitor technical performance. Additionally, the temporal distribution of user interactions was examined, and the conversation content was classified into thematic categories and communicative tone, enabling the characterization of usage patterns and the identification of user-expressed needs and preferences.

Ethical considerations. The study was approved and endorsed by the Ethics Committee of the Faculty of Nursing of the Universidad Autónoma de Nuevo León (No. FAEN-D-2021). It was conducted in accordance with strict ethical principles, ensuring data confidentiality and the voluntary participation of adolescents, following the prior obtainment of informed consent and assent.

## Results

### Study population description

The sociodemographic data showed that 49.6% of the participants were male and 50.4% were female. The mean age was 12.69 years (SD = 0.98), ranging from 11 to 15 years. By grade level, participants were distributed as follows: 32.4% were in the first year of secondary school, 36.5% in the second year, and 31.1% in the third year. This demographic distribution shows a balanced sample in terms of sex and is representative of the different levels of secondary education.

### “*Be Yourself*” chatbot metrics

**Figure 3:**
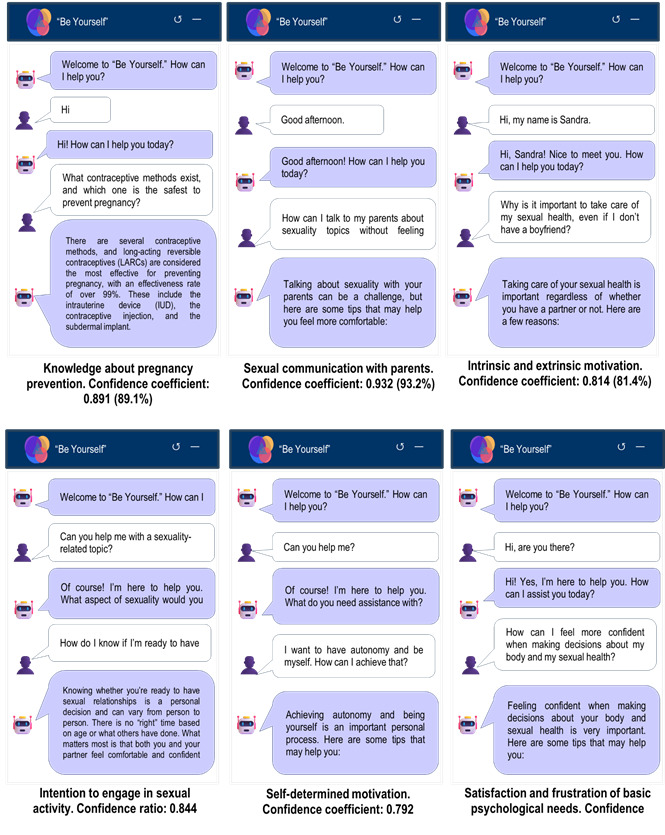
Chatbot-user conversations, with response confidence coefficients. Source: “*Be Yourself*” chatbot metrics from Chatbase.com

During the period evaluated, from August 18, 2024, to February 6, 2025, the “Be Yourself” chatbot recorded 74 chats and 1,236 messages exchanged. User interaction showed overall positive feedback, with 397 messages receiving a thumbs-up rating, suggesting high satisfaction, and only one message receiving a thumbs-down rating, indicating minimal dissatisfaction. Chat distribution usage showed a gradual post-intervention increase, with notable peaks in December 2024 (45 chats) and January 2025 (63 chats). These metrics indicate the utility of the chatbot as a support tool for adolescent sexual and reproductive health, addressing topics such as pregnancy prevention, sexual communication, self-determined motivation, satisfaction/frustration of BPNs, and intention to engage in sexual activity (Figure 3).

A total of 98 questions were identified that adolescents asked while using the chatbot. They consulted topics related to contraceptive methods (28.6%), followed by relationships and consent (19.4%) and sexually transmitted infections (STIs) (16.3%), reflecting interest in both pregnancy prevention and the emotional and social aspects of sexuality. Topics such as pregnancy (7.1%) and anatomy (3.1%) were less frequent, suggesting possible gaps in knowledge. Regarding interaction tone, most interactions involved requests for objective information (61.2%), although 19.4% express anxiety or social pressure, 9.2% presented urgent situations, and 4.1% reflected myths. This indicates that the chatbot not only serves an informational role but also provides emotional support and corrects misinformation.

According to chatbot-user interactions metrics, high confidence levels were observed across the different evaluated components of the “*Be Yourself*” program. The highest confidence coefficients were recorded in sexual communication with parents (93.2%), followed by knowledge about pregnancy prevention (89.1%) and satisfaction/frustration of BPNs (87.9%). Regarding motivational aspects, intrinsic and extrinsic motivation reached a confidence level of 81.4%, while self-determined motivation showed a slightly lower coefficient (79.2%). The component related to intention to engage in sexual activity showed a confidence level of 84.4%, placing it in an intermediate range among the different evaluated dimensions.

Post-intervention usability of the “Be Yourself” website. Most participants reported a high level of satisfaction with the website, and 97.3% reported liking it. Additionally, 79.7% said they would use it frequently (48.6% strongly agree and 31.1% agree), reflecting broad acceptance of the platform. Regarding ease of use, 93.3% perceived the website as easy to use (64.9% strongly agreed and 28.4% agreed), highlighting its intuitive and accessible interface. Similarly, 73% agreed that the website was very popular. In terms of perceived security, 79.8% of participants felt safe interacting with the website, and 89.2% agreed that its features were well integrated, indicating a coherent and functional structure. Regarding learnability, 86.5% believed that anyone could quickly learn to use the site, and the same percentage indicated they could use it without technical assistance, highlighting its self-contained, user-friendly design. Finally, regarding site consistency, 63.5% agreed that it was consistent, although 4.1% considered this consistency might be excessive. Additionally, 75.7% reported they could use the site without needing to learn anything new, confirming its intuitive and user-friendly design for users with no prior experience.

**Usability score*.*** Results showed that, in the initial post-intervention assessment (n=74), most participants rated system usability as “Acceptable” (81.1%, 60 cases), while 18.9% (14 cases) rated it as “Good.” The median and mode were 2, with scores ranging from 2 to 3 on the scale and a mean of 2.19. After two months of follow-up (*n*=70), the median remained at 2.00, but some changes in distribution were observed: 92.9% of participants (65 cases) continued to rate usability as “Acceptable,” while only 5.7% (4 cases) rated it as “Good.” Additionally, a “Poor” rating appeared for the first time (1 case, 1.4%). The mean decreased slightly to 2.04, and the range expanded (1 to 3). It should be noted that four cases were lost to follow-up (5.4% of the initial sample). Although essential measures remained stable, there was a notable decrease in positive ratings (“Good”) and the emergence of a negative rating (“Poor”) that had not been present in the initial measurement.

Before conducting the inferential analysis, the normality of the paired differences between post-test and follow-up SUS scores was assessed using the Shapiro-Wilk test. The results indicated a non-normal distribution (W = 0.92, p < 0.05), justifying the use of non-parametric tests. The Wilcoxon signed-rank test was applied to 70 complete data pairs (excluding 4 cases lost to follow-up, 5.4% of the initial sample, N = 74). The results showed a statistically significant decrease in SUS scores between the post-intervention assessment and the two-month follow-up (Z = -4.68, p < 0.001). The median decreased from 62.5 (IQR: 60.0-65.6) to 57.5 (IQR: 52.5-62.5). The effect size indicated a moderate-to-large reduction in perceived usability (r = -0.56). These findings may be related to reduced engagement with the chatbot, the need to updates its interactive design, or contextual changes in user needs during the monitoring period.

## Discussion

The study on the usability of the “Be Yourself” website and its AI-powered chatbot supports the growing role of digital interventions in adolescent sexual and reproductive health, demonstrating high initial acceptability. This is consistent with findings reported internationally, such as those from India, where 93% of adolescents reported positive evaluations of a similar chatbot due to its accessibility and comprehensive content, ^25^ and from Côte d'Ivoire, where 94% of users highlighted its ease of use.^26^ However, the significant decrease in perceived usability scores during the two-month follow-up reveals a critical challenge already documented in the literature: sustaining long-term user engagement. This limitation is consistent with findings from the SnehAI chatbot in India, which required ongoing content updates and personalization strategies to maintain engagement,^9^ as well as US studies emphasizing the need to adapt platforms in response to user feedback.^27^^,^^28^ These findings suggest that, although chatbots are promising tools for expanding access to sensitive information, their design must evolve toward more dynamic and interactive models to overcome user retention barriers, a key aspect for maximizing their impact on preventing adolescent pregnancy.^9^^,^^29^

The effectiveness of the chatbot in addressing specific topics, such as parent-adolescent communication on sexuality and pregnancy prevention, reflects its potential to address critical areas of adolescent health.^30^ However, lower confidence levels in complex psychological constructs, such as self-determined motivation, indicate limitations in AI's capacity to address emotional and motivational aspects.^31^ These findings support the position of several authors emphasizing the importance of complementing these technological tools with human interaction to achieve a comprehensive approach.^28^^,^^10^ The study also highlights the importance of culturally responsive design tailored to local needs as a key factor in the success of digital sexual health interventions.^15^^,^^25^^,^^32^^,^^33^ Although the chatbot demonstrated an entirely local user base (with 100% of interactions originating from Mexico), the lack of geographic diversity in the sample limits the generalizability of the results.^14^ This limitation is consistent with findings from similar studies conducted in other regional contexts, which emphasize that the effectiveness and acceptability of digital sexual health interventions are strongly influenced by cultural factors and require designs closely tailored to the target population.^34^^,^^35^ This underscores the need to include more diverse populations in future research. Compared with other interventions, such as CyberRwanda^33^ and Amanda Selfie^13^, the analysis identifies meaningful patterns in digital interventions for adolescent health. The findings indicate that, although they share core usability and accessibility features, “Be Yourself” stands out for its focus on self-determination theory, demonstrating greater effectiveness in sustained behavioral change.^18^ This difference highlights the importance of adapting interventions to specific local contexts, as recommended by studies conducted in Africa and Latin America to reduce adolescent pregnancy.^30^

Limitations. This study focused on perceived usability, measured using the SUS. Although this is a robust and standardized metric for assessing usability, future research could complement these findings with objective behavioral outcomes (changes in safer sexual behaviors or adherence to recommendations) and explore the potential of technologies such as generative AI to enhance personalization and intervention effectiveness.^36^^,^^37^ Finally, the “Be Yourself” chatbot represents a promising tool for preventing adolescent pregnancy, but its long-term success will require enhanced, sustained user engagement, integration with human-delivered services, and continuous adaptation to users' evolving needs through iterative refinement of the AI model.^38^ These findings reinforce the need to address adolescent pregnancy through a multifaceted approach combining technology, education, and psychosocial support, as recommended by the European Academy of Pediatrics.^39^

Implications for educational practice and implementation. The implementation of the “Be Yourself” chatbot in educational settings demonstrates its potential to support sexual health education through an innovative, accessible approach. Its accessible and confidential format can complement formal programs by facilitating adolescents’ access to information. These findings contribute to nursing knowledge by highlighting the value of digital tools for reaching adolescent populations and by offering a confidential channel that complements in-person interventions. However, the decrease in perceived usability over time suggests the need to complement these technologies with in-person educational strategies, regular feedback, and professional support that addresses adolescents’ emotional and motivational needs. In this sense, AI-driven adaptive personalization, together with cultural and linguistic tailoring, is a key component for maintaining engagement and supporting sustained behavior change effectively. For practice, these findings underscore the importance of integrating such technologies into multidisciplinary strategies, in which nursing professionals can play a central role in learning facilitation, educational reinforcement, and emotional support, leading professional monitoring, adaptive personalization, and cultural tailoring within an ethical and health promotion framework.^40^ Finally, the implementation of these interventions must be aligned with ethical and regulatory frameworks that ensure privacy, informed consent, and the responsible use of technology, promoting safe environments for digital and cross-sector learning. In this sense, the results not only advance knowledge about digital health interventions but also provide concrete guidelines for strengthening clinical and educational nursing practice in school and community settings.^41^

Conclusions. The study showed that the “Be Yourself” website and its AI-based chatbot represent an innovative and well-accepted alternative for addressing adolescent pregnancy prevention, fulfilling its goal of providing accessible and confidential information. However, the results also showed that, although these digital tools are effective in delivering targeted knowledge and facilitating sexual health communication, their scope remains primarily informational, meaning that addressing more complex psychosocial dimensions requires complementary strategies. The research confirms the value of technological interventions as complements rather than substitutes for comprehensive sexual health strategies for adolescents and highlights the need to integrate them into broader action frameworks that include educational, community, and public policy components. The findings provide relevant evidence for the development of future digital interventions in this field, highlighting the importance of balancing technological innovation with cultural relevance and adaptation to the real needs of the target population. Since usability was assessed post-intervention, the findings reflect the experience of using this system following initial implementation, an approach recommended for pilot studies of digital health interventions.
